# Methods of causal effect estimation for high‐dimensional treatments: A radiotherapy simulation study

**DOI:** 10.1002/mp.17919

**Published:** 2025-06-02

**Authors:** Alexander Jenkins, Eliana Vasquez Osorio, Andrew Green, Marcel van Herk, Matthew Sperrin, Alan McWilliam

**Affiliations:** ^1^ Department of Electrical and Electronic Engineering Imperial College London London UK; ^2^ Division of Cancer Sciences University of Manchester Manchester UK; ^3^ The Christie NHS Foundation Trust Manchester UK; ^4^ European Bioinformatics Institute (EMBL‐EBI) Cambridge UK; ^5^ Division of Informatics Imaging & Data Sciences University of Manchester Manchester UK

**Keywords:** causal inference, radiotherapy outcome modelling, voxel‐based analysis

## Abstract

**Background:**

Radiotherapy, the use of high‐energy radiation to treat cancer, presents a challenge in determining treatment outcome relationships due to its complex nature. These challenges include its continuous, spatial, high‐dimensional, multi‐collinear treatment, and personalized nature, which introduces confounding bias.

**Purpose:**

Existing voxel based estimators may lead to biased estimates as they do not use a causal inference framework. We propose a novel estimator using sparsity via Adaptive Lasso within Pearl's causal framework, the Causal Adaptive Lasso (CAL).

**Methods:**

First, simplified 2‐dimensional treatment plans were simulated on 10×10 and 25×25 grids. Each simulation had an organ at risk placed in a consistent location where dose was minimized and a randomly placed target volume where dose was maximized. Treatment uncertainties were simulated to emulated a fractionated delivery. A directed acyclic graph was devised which captured the causal relationship between our outcome, including confounding.

The estimand was set to the associated dose‐outcome response for each simulated delivery (n=500). We compared our proposed estimator the CAL against established voxel based regression estimators using planned and delivered simulated doses. Three variations on the causal inference‐based estimators were implemented: causal regression without sparsity, CAL, and pixel‐wise CAL. Variables were chosen based on Pearl's Back‐Door Criterion. Model performance was evaluated using Mean Squared Error (MSE) and assessing bias of the recovered estimand.

**Results:**

CAL is tested on simulated radiotherapy treatment outcome data with a spatially embedded dose response function. All tested CAL estimators outperformed voxel‐based estimators, resulting in significantly lower total MSE, ${\text{MSE}}_{tot}$, and bias, yielding up to a four order of magnitude improvement in ${\text{MSE}}_{tot}$ compared to current voxel‐based estimators (${\text{MSE}}_{tot}<1\times 10^{2}$ compared to ${\text{MSE}}_{tot}\approx 1\times 10^{6}$). CAL also showed minimal bias in pixels with no dose response.

**Conclusions:**

This work shows that leveraging sparse causal inference methods can benefit both the identification of regions of given dose‐response and the estimation of treatment effects. Causal inference methodologies provide a powerful approach to account for limitations in voxel‐based analysis. Adapting causal inference methodologies to the analysis of clinical radiotherapy treatment‐outcome data could lead to new and impactful insights on the causes of treatment complications.

## 1 INTRODUCTION

A recent focus of radiotherapy research has been to identify dose‐sensitive regions of the anatomy from observational data, whereby the voxel‐based analysis has emerged as the de‐facto method.^2^ In this method, researchers regress the continuous planned radiation dose at each voxel against the observed complication outcomes, to map spatial dose response relationships. The voxel‐based analysis has been applied to several treatment sites, including the bladder,^3^ parotid glands,^4^ the lung,^5^ the heart^6^ and has also been adapted for the study of treatment errors on outcomes.^7^

While the voxel‐based analysis has been effective in mapping spatial dose response relationships, it does not adequately control for the confounding bias induced by the personalized nature of radiotherapy treatment. As a result, its ability to establish causal relationships between dose and complications is limited. In fact, determining causal relationships from observational data is impossible outside of a causal inference framework.

The Average Treatment Effect (ATE) is a common estimand in causal inference literature, whereby a treatment A is said to have a finite ATE on the outcome Y if $E\left[Y^{A=a_{1}}\right]\neq E\left[Y^{A=a_{2}}\right]$, where $a_{1}$ and $a_{2}$ represent exposure versus no exposure, two competing treatments, or the same treatment modality but at different doses. The task of ATE estimation is typically addressed through Randomized Controlled Trials (RCTs), where random assignment balances treatment and control groups for unbiased ATE estimation. However, RCTs may not always be feasible or ethical, such as in radiotherapy, where dose must be personalized.

To estimate ATEs when RCTs cannot be conducted, alternative causal frameworks must be employed. For instance, Pearl's Structural Causal Models (SCMs) and “do‐calculus”, can be employed to estimate ATEs using observational data.^8^ In Pearl's framework, Direct Acyclic Graphs (DAGs) represent the conditional independence properties between variables, and the SCM is the system of generative equations that relate the variables together. ATE estimation is then possible using SCMs by leveraging Pearl's adjustment criteria and do‐calculus in the form of an outcome/causal regression. However, these techniques have not yet been explored for high‐dimensional and spatially complex treatments, such as radiotherapy.

This work proposes a novel causal inference estimator, *Causal Adaptive Lasso (CAL)*, for estimating causal effects between each component of a continuous, spatial, high‐dimensional, and multi‐collinear treatment, and an outcome variable. Specifically, CAL leverages the sparsity‐inducing properties of the Adaptive Lasso^9^ within Pearl's causal framework to achieve two key objectives: 1) efficient estimation, by reducing the dimensionality of the adjustment set via sparsity; and 2) multi‐collinearity control, by incorporating a ridge regression step in the estimation of Adaptive Lasso weights.

To evaluate CAL, a simulation study is conducted to generate radiotherapy treatment outcome data from a simplified, yet realistic, data‐generating process. This study serves as a first introduction of causal inference within the context of the radiotherapy voxel‐based analysis. The simulation will investigate if CAL: 1) outperforms existing voxel‐based estimators across various simulation settings; 2) can effectively identify voxels with zero dose response due to the sparsity mechanism; and 3) can scale to treatments with higher resolution/dimensionality.

## 2 METHODS

2.1

Our simulation aims to investigate what conditions are required for unbiased voxel‐based causal inference. Specifically, we are interested in quantifying the performance of our estimation methods when the following are changed: data set size, resolution of the treatment plan (number of pixels), and the size of random treatment uncertainties in treatment delivery. To do this, we simulate data from a simplified, yet realistic, data‐generating process for radiotherapy treatment outcome data.

### 2.2 Data‐generating process

**FIGURE 1:**
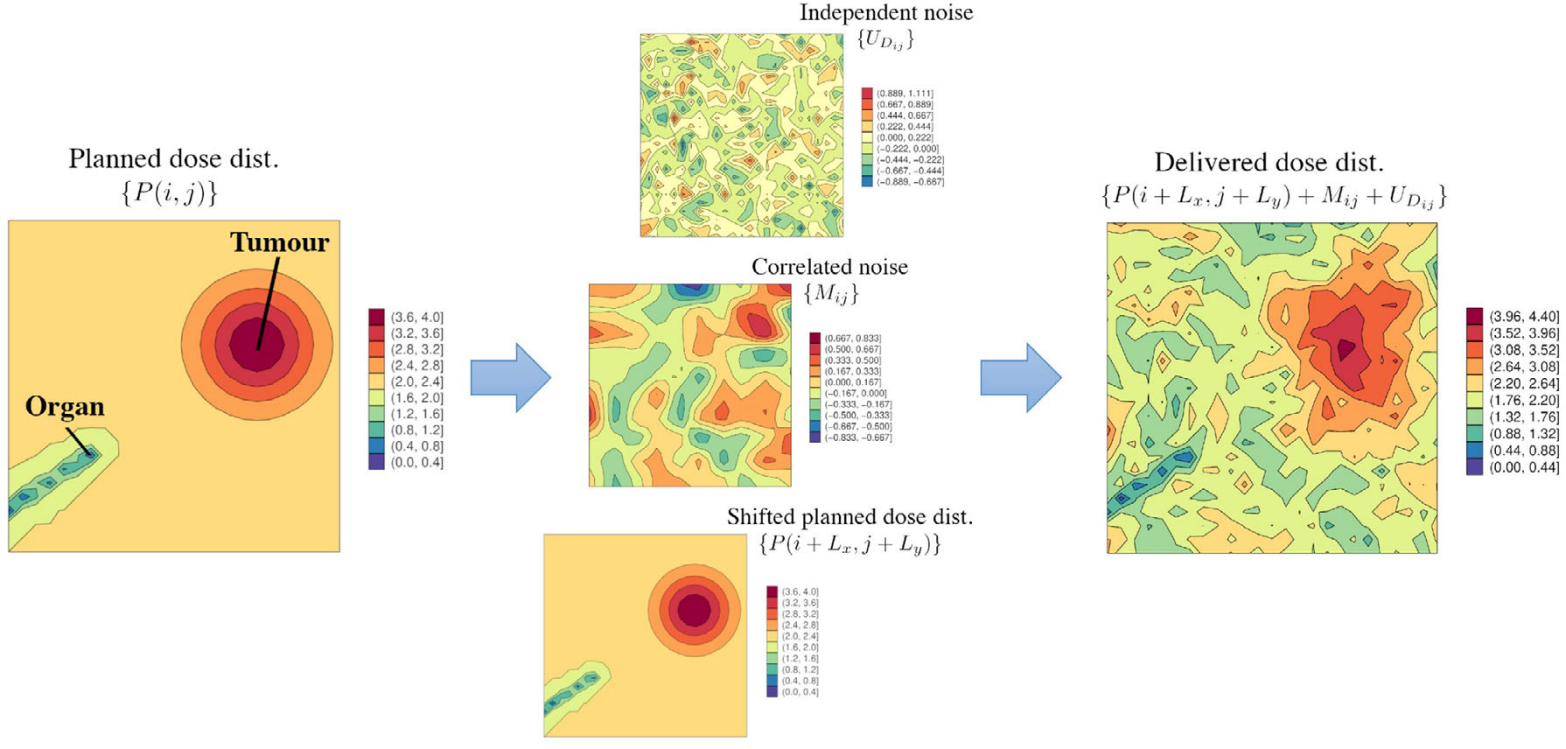
An example of a simulated planned and delivered dose distribution for a patient. To simulate realistic delivered dose distributions, three different treatment uncertainties are modeled: dosimetric uncertainty (independent white noise), anatomical motion (correlated noise via Gaussian process), and setup uncertainty (shifted planned dose distribution).

Patients were modeled on 2D grids (10×10 or 25×25 pixels) with a fixed OAR location and randomly placed tumor. Treatment plans were simulated to maximize tumor dose while minimizing OAR dose, following VMAT treatment principles.^10^ Delivered dose distributions incorporated three realistic treatment uncertainties: dosimetric uncertainty, anatomical motion, and setup uncertainty (Figure 1). Detailed simulation methods are provided in Supplementary Materials S.1.A‐B.

#### 2.2.1 DAG and SCM

**FIGURE 2:**
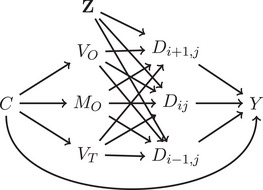
The causal structure between variables used in this simulation, represented using a DAG. Variable C is a measured confounder, $V_{O}$ is a variable that controls the fall‐off of dose around the organ ray, $M_{O}$ is a variable that controls the magnitude of dose on the organ ray, $V_{T}$ is a variable that controls the fall‐off of dose around the tumor, $D_{ij}$ is the value of the delivered dose distribution at the pixel location x=i,y=j, and Z is a latent variable representing the parameters of the treatment uncertainties. The DAG is drawn only for three pixels, but extends over all pixels in this work.

The causal structure used in our simulation is shown in Figure 2. The dose distribution D is determined by clinical parameters controlling dose fall‐off ($V_{O}$, $V_{T}$) and magnitude ($M_{O}$) around organs and tumors, along with treatment uncertainties represented by latent variable Z. A patient covariate C (e.g., concurrent chemotherapy) personalizes treatment by affecting $V_{O}$, $M_{O}$, and $V_{T}$, while also confounding the relationship between treatment and outcome. For instance, covariate C could represent concurrent chemotherapy dosage, which could affect the treatment plan and the treatment outcomes.^11^

**FIGURE 3:**
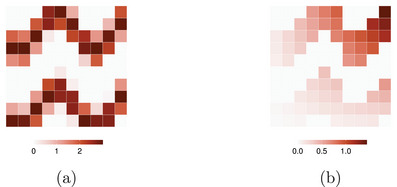
(a) The estimand array (produced using supplementary Equation 19), and (b) the interaction strength array (produced using supplementary Equation 20), where the number of pixels in the x‐ and y‐directions is equal to $N_{x}=10$ and $N_{y}=10$, respectively.

Variables were generated using a SCM with non‐linear functions for C, $V_{T}$, $V_{O}$ and $M_{O}$ (detailed in Supplementary Materials S.1.C). The outcome Y was modeled linearly as

(1) $$Y=5C+U_{Y}+\sum_{i=1}^{N_{x}}\sum_{j=1}^{N_{y}}\left(D_{ij}\theta_{ij}+\frac{C}{2}\left(D_{ij}\xi_{ij}\right)\right),$$

where θ and ξ represent sparse, inhomogeneous dose response and interaction strength arrays, respectively, as shown in Figure 3 (details in Supplementary Materials S.1.D). This linear outcome model was chosen for clarity in introducing causal inference concepts, compatibility with existing voxel‐based methods, and to enable fair comparison between estimators.

### 2.3 Estimands

Our chosen estimand is the ATE of delivered dose $D_{ij}$ on outcome Y at each pixel. Given the form of Equation (1), this coincides with the Conditional Average Treatment Effect (CATE) at C=0, CATE(C=0). While both estimands share the same expectation θ, they differ in variance: unconditional effects have variance $\xi_{ij}^{2}/4$ while conditional effects have zero variance (derivation in Supplementary Materials I.C). This variance difference represents a trade‐off between estimation precision and population‐level generalizability; conditioning on C improves accuracy but may limit generalizability to broader populations.

### 2.4 Estimation methods

We compare our proposed CAL estimator with two established voxel‐based approaches: pixel‐wise planned dose and pixel‐wise delivered dose regressions (detailed in Supplementary Materials S.2). We also evaluate two additional causal estimators: pixel‐wise CAL and causal regression without sparsity. All causal methods share four key assumptions: (1) known causal structure (Figure 2), (2) SCM‐based data generation, (3) linear treatment effects on outcomes, and (4) conditional ignorability, causal consistency, and positivity.

These five estimation methods were carefully selected to demonstrate the impact of causal inference and sparsity in voxel‐based analysis. The pixel‐wise planned and pixel‐wise delivered dose regressions represent current state‐of‐the‐art approaches in radiotherapy literature. The causal regression without sparsity isolates the contribution of the causal framework alone, while CAL and pixel‐wise CAL demonstrate the combined benefits of causal inference with sparsity‐inducing regularization. This comparative framework allows us to quantify performance improvements attributable to each methodological component.

To achieve unbiased causal effect estimation, adjustment sets were identified using the Back‐Door criterion,^12^ which isolates variables that block all backdoor paths between treatment and outcome. The adjustment sets for ATE inference at pixel (i,j) are given by

(2a) $$\begin{array}{cc} A & =\{D-\left\{D_{ij}\right\},C\}; \end{array}$$

(2b) $$\begin{array}{cc} A & =\{D-\left\{D_{ij}\right\},V_{O},M_{O},V_{T}\}. \end{array}$$

Where $D-\{D_{ij}\}$ represents all values in dose distribution except at the current pixel. We selected adjustment set (2 b) for our simulation as it provides greater robustness against potential direct effects of $M_{O}$, $V_{O}$ and $V_{T}$ on the outcome. This adjustment set, in addition to $D_{ij}$, forms the total set of features that will be used for ATE estimation at each pixel.

#### The Causal Adaptive Lasso (CAL)

Our proposed estimator, CAL, employs a linear Adaptive Lasso with the identified adjustment set as features. We selected Adaptive Lasso for its oracle property (consistency in variable selection and parameter estimation) and its ability to handle the sparse nature of dose response.^9^ This sparsity facilitates identification of critical anatomical regions by reducing treatment variable dimensionality. The Adaptive Lasso parameters are estimated by

(3) $$\hat{\beta }=\underset{\beta }{arg min}\left(\sum_{i=1}^{n}{\left(y_{i}-\sum_{j=1}^{p}X_{ij}\beta_{j}\right)}^{2}+\lambda \sum_{j=1}^{p}w_{j}\left|\beta_{j}\right|\right),$$

where n is the training data size, p is the feature count, $X_{ij}$ represents elements of the training data matrix, and $w\in R^{p}$ is the adaptive weight vector.^13^ These weights are calculated as

(4) $$\hat{w}=\frac{1}{|{\hat{\beta }}^{*}{|}^{\gamma }},$$

with γ=1 for simplicity and ${\hat{\beta }}^{*}$ derived from ridge regression to mitigate multi‐collinearity between pixels.^14^ Adaptive weights for $V_{O}$, $M_{O}$ and $V_{T}$ are set to zero to ensure their inclusion in the model. Both ridge and Adaptive Lasso hyperparameters ($\lambda_{ridge}$ and λ) are selected through 10‐fold cross‐validation using glmnet ^15^ in r ^16^ to minimize MSE.

We also test pixel‐wise CAL, which applies the same approach at each individual pixel (resulting in $N_{x}N_{y}$ separate regressions) and sets the adaptive weight for the current pixel's dose ($D_{ij}$) to zero, hypothesizing this may reduce estimation bias.

While our simulation study focuses on the 2D case for simplicity, CAL is designed to scale to 3D dose distributions. The sparsity‐inducing properties of the Adaptive Lasso provide an inherent advantage when moving to higher dimensions, as it identifies only those voxels with significant causal effects, effectively reducing dimensionality. This is particularly important for radiotherapy applications, where the curse of dimensionality becomes more pronounced when transitioning from 2D, $O(N^{2})$ pixels, to 3D, $O(N^{3})$ voxels, where N represents the number of pixels or voxels along each dimension. Meanwhile, the number of available patient observations, $n_{obs}$, typically remains limited in clinical settings.

#### Computational Complexity

The computational complexity of these estimation methods varies considerably. Computing the closed‐form solution for each linear regression is dominated by matrix inversion, which has a time complexity of $O(p^{3})$. Pixel/voxel‐wise approaches require solving approximately $N^{d}$ separate regressions, where d represents the dimensionality of the dose distribution (e.g., d=2 for 2D), resulting in a worst‐case complexity of $O(p^{3}N^{d})$. In contrast, CAL and causal regression without sparsity involve a single regression with substantially higher feature dimensionality (approximately $N^{d}$ features), resulting in a higher complexity of $O(N^{3d})$. While appearing formidable, gradient descent is often employed instead of finding the closed‐form solution to achieve better scalability. For CAL, gradient descent would significantly reduce the computational complexity to $O(mnN^{d})$, where m is the number of gradient descent steps, making it more similar to the complexity order of pixel/voxel‐wise approaches.

### 2.5 Performance measures

For each estimation method, Monte Carlo estimates of the ATE at each pixel will be calculated as ${\hat{\theta }}_{ij}=1/n_{sim}\sum_{k=1}^{n_{sim}}{\hat{\theta }}_{ijk}$, where ${\hat{\theta }}_{ijk}$ is the ATE estimate at each pixel for the $k^{th}$ repetition of the simulation. This will be used to form Monte Carlo estimate arrays, $\hat{\theta }=\left\{{\hat{\theta }}_{ij}\right\}$. This will be repeated for each possible combination of simulation parameters shown in Table 1.

**TABLE 1:** The table of parameter values to be tested in this simulation.

Parameters	Values to test
$n_{obs}$	50, 100, 500
$N_{x},N_{y}$	10, 25
$\sigma_{U_{D_{ij}}}^{2}$	3e−12, 3e−2, 3e−1, 3
$M_{GP}$	3e−12, 3e−2, 3e−1, 3
$\sigma_{GP}^{2}$	3e−12, 3e−2, 3e−1, 3
$\sigma_{L}^{2}$	3e−12, 3e−2, 3e−1, 3

*Note*: Each possible combination of these parameters will be tested for all estimations methods. The parameter $n_{obs}$ refers to the number of patients simulated, $N_{x},N_{y}$ is the number of pixels in the x‐ and y‐directions, respectively, $\sigma_{U_{D_{ij}}}^{2}$ is the variance of the independent noise at each pixel (held constant across all pixels), $M_{GP}$ is the magnitude of the Gaussian process, $\sigma_{GP}^{2}$ is the variance of the radial basis function kernel used in the Gaussian process, and $\sigma_{L}^{2}$ is the variance used to generate $L_{x}$ and $L_{y}$; set‐up uncertainty shifts in x‐ and y‐directions.

As the interest is in whether the estimation method can make unbiased estimates of θ, bias is selected as a performance measure. Specifically, the bias at each pixel will be calculated and visualized. The bias array is defined as $B=\{B_{ij}|i\in \left[1,\text{…},N_{x}\right],j\in \left[1,\text{…},N_{y}\right]\}$ where,

(5) $$B_{ij}=E\left[{\hat{\theta }}_{ij}\right]-\theta_{ij}\approx \frac{1}{n_{sim}}\sum_{k=1}^{n_{sim}}{\hat{\theta }}_{ijk}-\theta_{ij}.$$

As a global metric to compare different simulation setups, the sum of MSE across all pixels, ${\mathrm{MSE}}_{tot}$, will be used. This is defined as

(6) $${\mathrm{MSE}}_{tot}=\sum_{i=1}^{N_{x}}\sum_{j=1}^{N_{y}}{\mathrm{MSE}}_{ij},$$

where,

(7) $${\mathrm{MSE}}_{ij}=E\left[{\left({\hat{\theta }}_{ij}-\theta_{ij}\right)}^{2}\right]\approx \frac{1}{n_{sim}}\sum_{k=1}^{n_{sim}}{\left({\hat{\theta }}_{ijk}-\theta_{ij}\right)}^{2}.$$

These performance metrics were selected for their relevance to causal effect estimation in radiotherapy. ${\mathrm{MSE}}_{tot}$ provides a comprehensive global assessment capturing both bias and variance components across all pixels, while the bias array offers spatial insight into systematic estimation errors. This combination evaluates both overall performance and location‐specific accuracy, which is crucial for identifying anatomical regions with dose‐response relationships. While alternative metrics such as Mean Absolute Error (MAE) or sparsity recovery metrics (precision/recall) could be employed, our chosen metrics directly assess the primary goal of voxel‐based analysis: accurate estimation of continuous causal effects across the spatial domain.

## 3 RESULTS

**FIGURE 4:**
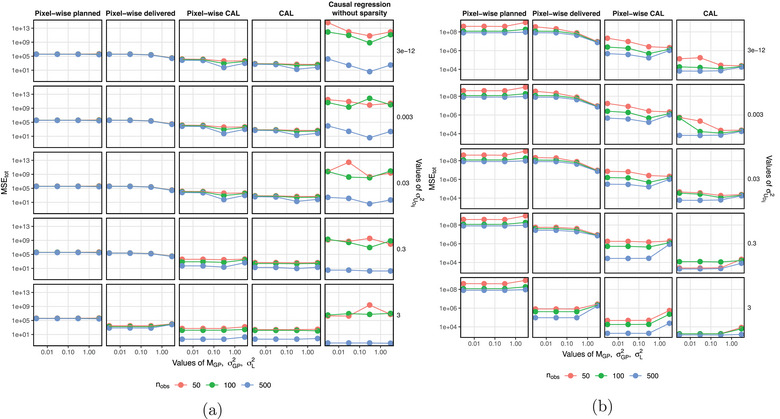
Line plots of ${\mathrm{MSE}}_{tot}$ against values of $M_{GP}$, $\sigma_{GP}^{2}$ and $\sigma_{L}^{2}$ for (a) $N_{x},N_{y}=10$ and (b) $N_{x},N_{y}=25$. Colors represent values of $n_{obs}$. The rows represent different values of $\sigma_{U_{D_{ij}}}^{2}$, with the exact value labeled. The columns represent the results of the estimation methods.

Figure 4a,b shows the global performance, ${\text{MSE}}_{tot}$, of each estimation method (1) pixel‐wise planned, (2) pixel‐wise delivered, (3) pixel‐wise CAL, (4) CAL and (5) the causal regression without sparsity; where (1) and (2) represent state‐of‐the‐art voxel‐based methods. ${\text{MSE}}_{tot}$ is shown for different values of sample sizes ($n_{obs}$) and treatment uncertainty parameters for dosimetric uncertainty ($\sigma_{U_{D_{ij}}}^{2}$), anatomical motion ($M_{GP}$ and $\sigma_{GP}^{2}$), and setup uncertainty ($\sigma_{L}^{2}$), where Figure 4a,b shows results at a low resolution of 10×10 pixels and a higher resolution of 25×25 pixels, respectively.

Across all parametrizations of sample sizes and treatment uncertainties in Figure 4a,b, we find that all CAL‐based estimators achieve consistently lower ${\text{MSE}}_{tot}$ than pixel‐wise planned and pixel‐wise delivered methods. This is highlighted by performances of ${\text{MSE}}_{tot}<1\times 10^{2}$ for 10×10 resolution, compared to approximately $1\times 10^{6}$ for pixel‐wise planned and pixel‐wise delivered methods; and performances of ${\text{MSE}}_{tot}$ between $1.3\times 10^{3}$ and $1\times 10^{5}$ for 25×25 resolution, compared to approximately $1\times 10^{8}$ for pixel‐wise planned and $1\times 10^{7}$ for pixel‐wise delivered methods. The best‐performing CAL and pixel‐wise CAL estimators achieved ${\text{MSE}}_{tot}=0.450$ and ${\text{MSE}}_{tot}=0.452$ for 10×10 resolution, respectively, and ${\text{MSE}}_{tot}=1323.3$ and ${\text{MSE}}_{tot}=2016.2$ for 25×25 resolution, respectively. This represents a consistent improvement of approximately four orders of magnitude across both resolutions.

This shows that the increasing number of pixels has had an effect on accuracy. At a resolution of 10×10 pixels, the causal regression without sparsity performed with the lowest ${\text{MSE}}_{tot}$ of all methods when $n_{obs}=500$ and $\sigma_{U_{D_{ij}}}^{2}=3$. However, the global performance of the causal regression without sparsity displayed a strong dependence on $n_{obs}$ and treatment uncertainties, whereas the methods with sparsity did not. In addition, the causal regression without sparsity failed to converge at the higher resolution of 25×25 pixels and is therefore not included in Figure 4b. This can be attributed to the absence of sparsity regularization and the lack of control over multi‐collinear features within the adjustment set. On the other hand, CAL performed with consistently low ${\text{MSE}}_{tot}$ and showed a little drop in global performance across all parametrizations of sample sizes and treatment uncertainties. This highlights the utility of a sparsity‐inducing Adaptive Lasso to estimate causal effects in the high‐dimensional setting.

**FIGURE 5:**
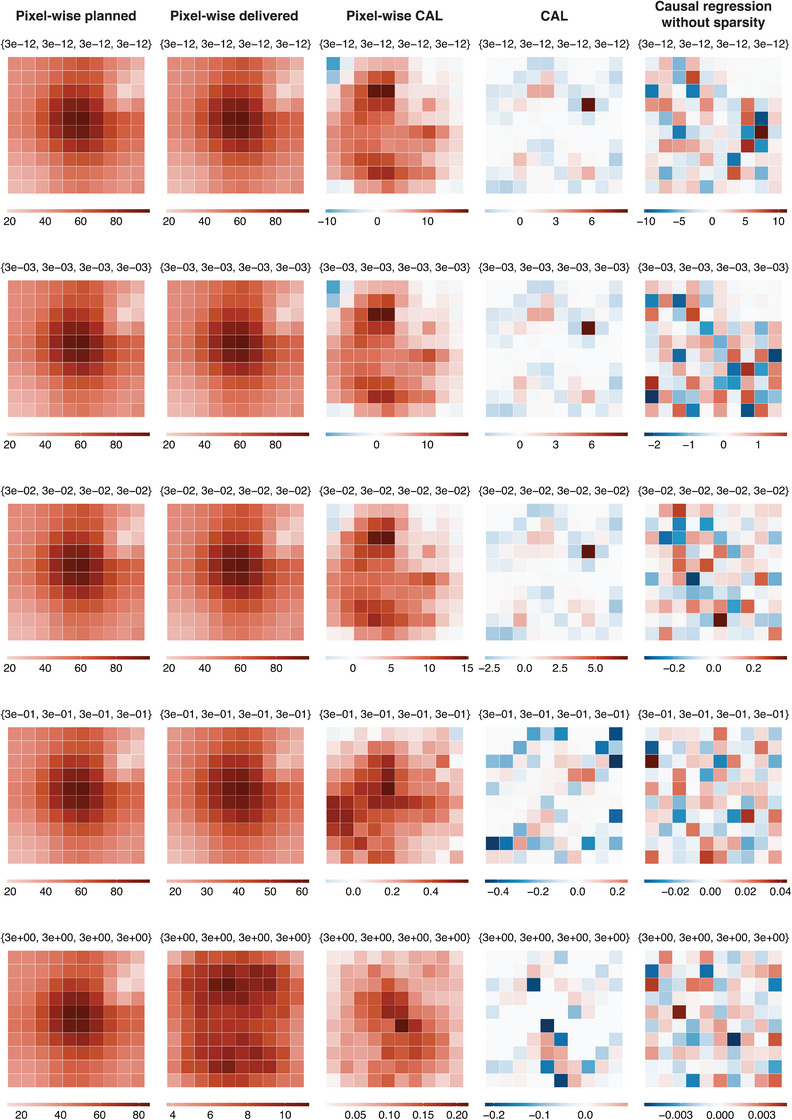
Visualizations of the Monte Carlo estimates of the bias array, B, of each estimation method for the simulation run with different parametrizations of $\sigma_{U_{D_{ij}}}^{2}$, $M_{GP}$, $\sigma_{GP}^{2}$ and $\sigma_{L}^{2}$, with values shown above each sub‐figure as $\left\{\sigma_{U_{D_{ij}}}^{2},M_{GP},\sigma_{GP}^{2},\sigma_{L}^{2}\right\}$. Results are for constant values of $n_{obs}=500$, and $N_{x},N_{y}=10$. The columns represent the results of the estimation methods.

**FIGURE 6:**
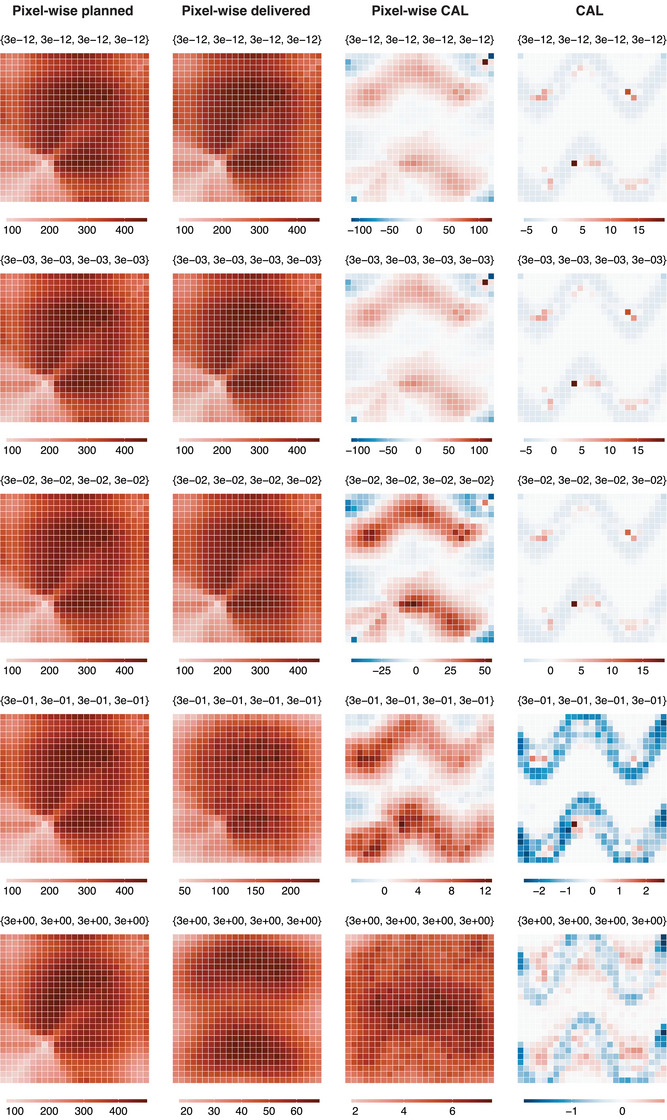
Visualizations of the Monte Carlo estimates of the bias array, B, of each estimation method for the simulation run with different parametrizations of $\sigma_{U_{D_{ij}}}^{2}$, $M_{GP}$, $\sigma_{GP}^{2}$ and $\sigma_{L}^{2}$, with values shown above each sub‐figure as $\left\{\sigma_{U_{D_{ij}}}^{2},M_{GP},\sigma_{GP}^{2},\sigma_{L}^{2}\right\}$. Results are for constant values of $n_{obs}=500$, and $N_{x},N_{y}=25$. The columns represent the results of the estimation methods.

Figure 5 represents the local performance as bias maps for each estimation method for $n_{obs}=500$ and across different parametrizations treatment uncertainties, at a resolution of 10×10 pixels. Firstly, it is observed that the pixel‐wise delivered method utilizing delivered dose can estimate the ATE with lower bias as treatment uncertainties increase. The bias at each pixel for the causal regression without sparsity reduces as treatment uncertainties increase. Our method, CAL, performs well over all parametrizations, unlike the causal regression without sparsity and the pixel‐wise CAL, where bias showed stronger dependencies on treatment uncertainties. In addition, when the treatment uncertainties are all set to $3\times 10^{-12}$, that is, when the delivered dose distribution is approximately equal to the planned dose distribution, CAL handles the high degree of multi‐collinearity between variables in the adjustment set (dose at adjacent pixels) well. This is unlike the causal regression without sparsity, where the bias map contains much larger values. Interestingly, CAL has a very low bias in the regions where the estimand is zero and a larger bias in the regions where the estimand is non‐zero; which could allow regions of dose response to be accurately identified. This reflects the Adaptive Lasso's oracle property of consistency in variable selection. However, utilization of the ridge regression in our method does bias parameter estimates where parameters are deemed important. The same observations hold for the bias maps produced by all estimation methods for $n_{obs}=500$ and 25×25 pixels, as shown in Figure 6.

These results demonstrate the substantial performance advantages of causal inference‐based approaches over traditional voxel‐based regression methods across multiple simulation settings. The comparative analysis across all five estimation methods shows that explicitly modeling the causal structure with appropriate adjustment sets leads to significantly improved estimation accuracy, with the sparsity‐inducing CAL methods maintaining consistent performance even at higher resolutions and with smaller sample sizes.

## 4 DISCUSSION

In this work, a sparse estimator (the Adaptive Lasso within Pearl's causal framework), CAL, has been proposed to estimate the treatment effects of each component of a high‐dimensional treatment space. The methodology successfully recovers unbiased, spatial, dose response functions from simulated radiotherapy data. Leveraging the Adaptive Lasso and its oracle properties in our exemplar problem from radiotherapy has allowed us to simultaneously identify regions within the high‐dimensional treatment with non‐zero dose response and estimate the ATE of the treatment components on an outcome. Our comparison of estimation methods reveals that conventional voxel‐based approaches, by ignoring confounding factors, fail to account for the personalized nature of radiotherapy treatment planning. CAL addresses this limitation while leveraging sparsity to improve estimation efficiency, particularly in higher‐dimensional settings.

Voxel‐based analysis originated in neuroimaging^17^ before being adapted to radiotherapy, with sparse statistical models following a similar path.^18^, ^19^ Whilst not a first introduction of causal inference to radiotherapy, this work is the first introduction of causal inference to the voxel‐based analysis. Previous causal approaches such as Nabi et al.'s^20^ are not applicable to voxel‐based analysis where features greatly exceed observations. Their approach of representing high‐dimensional treatments with low‐dimensional representations that preserve cause‐effect relationships faces interpretability challenges^21^ when mapping back to original features; a crucial requirement for identifying anatomical regions with dose response. Our sparse causal inference method offers new insights into causal mechanisms within complex imaging data while maintaining interpretability.

Our simulation captured key complexities of radiotherapy treatment outcome data, though several simplifying assumptions were made. We assumed no interaction between delivered dose values across voxels, consistent with concepts of functional sub‐units in organs introduced by Schultheiss et al.^22^ and Withers et al.^23^ We also assumed constant tumor and organ shapes, ignoring potential confounding from segmentation variations that could mediate clinical factors. While our SCM used linear outcome generation, clinical relationships are likely non‐linear, potentially requiring non‐parametric sparse methods like the Sparse Bayesian Causal Forest^24^ to capture heterogeneous treatment effects.

For real‐world application, our approach requires further development. Future work should incorporate additional clinical variables affecting delivered dose beyond $V_{O}$, $M_{O}$ and $V_{T}$, such as dose‐volume histogram statistics from multiple OARs. The delivered dose's random component ($U_{D_{ij}}$) enables causal inference and can be estimated using techniques like those of Shelley et al.^25^ For clinical data with more complex relationships, sensitivity analysis could verify the assumed DAG's appropriateness before identifying adjustment sets using Pearl's criteria. Extensions to handle segmentation‐based confounding may benefit from causal representation learning approaches,^26^ enabling CAL to analyze real patient data while accounting for these complexities.

Although demonstrated in 2D, extending CAL to 3D dose distributions is straightforward from a methodological perspective. For clinical implementation with full 3D datasets, computational efficiency could be enhanced through multi‐resolution approaches, where initial analysis at lower resolution identifies regions of interest for more detailed investigation. Additionally, anatomical knowledge could be incorporated through structured sparsity constraints, leveraging the fact that dose‐response relationships often occur in contiguous regions rather than isolated voxels. These extensions would preserve CAL's theoretical advantages while making it computationally feasible for the substantially higher dimensionality of full 3D clinical dose distributions.

CAL offers several key clinical benefits for radiotherapy: (1) more accurate identification of radiosensitive regions by accounting for confounding factors that conventional methods ignore; (2) improved treatment personalization through precise mapping of critical subregions; (3) potential identification of patient subgroups with differential sensitivity profiles; and (4) more reliable guidance for adaptive radiotherapy by distinguishing causal from correlative relationships. To realize these benefits, future work should apply CAL to clinical toxicity data, addressing additional factors including patient‐specific anatomical variations, inter‐observer segmentation variability, additional clinical covariates, and non‐linear dose‐response relationships. The significant performance improvements demonstrated in our controlled simulations provide a strong foundation for these clinical applications.

While this work focuses on causal effect estimation at Pearl's intervention level,^8^ future research could explore counterfactuals; the highest level of Pearl's causal ladder. Extending CAL to generate counterfactual outcomes for individuals would enable prediction under hypothetical interventions, potentially identifying optimal radiotherapy treatments for individual patients. This progression from intervention to counterfactual analysis, combined with extensions to full 3D dose distributions through multi‐resolution approaches and structured sparsity constraints, represents a promising direction for personalized radiotherapy that maintains CAL's theoretical advantages while addressing the computational challenges of higher‐dimensional clinical data.

## CONFLICT OF INTEREST STATEMENT

The authors declare no conflicts of interest.

## Supporting information


Supporting Information

## References

[1] Jenkins AL . Voxel‐Based Causal Inference in Radiotherapy: A Simulation Study. Master's thesis. University of Manchester; 2021.

[2] Palma G , Monti S , Cella L . Voxel‐based analysis in radiation oncology: a methodological cookbook. Physica Med. 2020;69:192‐204. 10.1016/j.ejmp.2019.12.013 31923757

[3] Improta I , Palorini F , Cozzarini C , et al. Bladder spatial‐dose descriptors correlate with acute urinary toxicity after radiation therapy for prostate cancer. Physica Med. 2016;32(12):1681‐1689. 10.1016/j.ejmp.2016.08.013 27570122

[4] Luijk vP , Pringle S , Deasy JO , et al. Sparing the region of the salivary gland containing stem cells preserves saliva production after radiotherapy for head and neck cancer. Sci Transl Med. 2015;7(305):305ra147‐305ra147. 10.1126/scitranslmed.aac4441 PMC4964284 26378247

[5] Seppenwoolde Y , Jaeger KD , Boersma LJ , Belderbos JS , Lebesque JV . Regional differences in lung radiosensitivity after radiotherapy for non–small‐cell lung cancer. Int J Radiat Oncol Biol Phys. 2004;60(3):748‐758. 15465191 10.1016/j.ijrobp.2004.04.037

[6] McWilliam A , Kennedy J , Hodgson C , Osorio EV , Faivre‐Finn C , Herk vM . Radiation dose to heart base linked with poorer survival in lung cancer patients. Eur J Cancer. 2017;85:106‐113. 28898766 10.1016/j.ejca.2017.07.053

[7] Jenkins A , Mullen TS , Johnson‐Hart C , et al. Novel methodology to assess the effect of contouring variation on treatment outcome. Med Phys. 2021;48(6):3234‐3242. 33772803 10.1002/mp.14865

[8] Pearl J . Causation, Action, and Counterfactuals. In: Logic and Scientific Methods. Springer; 1997:355‐375.

[9] Zou H . The adaptive Lasso and its oracle properties. J Am Stat Assoc. 2006;101(476):1418‐1429.

[10] Palma D , Vollans E , James K , et al. Volumetric modulated arc therapy for delivery of prostate radiotherapy: comparison with intensity‐modulated radiotherapy and three‐dimensional conformal radiotherapy. Int J Radiat Oncol Biol Phys. 2008;72(4):996‐1001. 18455326 10.1016/j.ijrobp.2008.02.047

[11] Forastiere AA , Goepfert H , Maor M , et al. Concurrent chemotherapy and radiotherapy for organ preservation in advanced laryngeal cancer. N Engl J Med. 2003;349(22):2091‐2098. 14645636 10.1056/NEJMoa031317

[12] Pearl J . Causal diagrams for empirical research. Biometrika. 1995;82(4):669‐688.

[13] Tibshirani R . Regression shrinkage and selection via the Lasso. J R Stat Soc (Series B). 1996;58:267‐288.

[14] Hoerl AE , Kennard RW . Ridge regression: biased estimation for nonorthogonal problems. Technometrics. 1970;12(1):55‐67.

[15] Friedman J , Hastie T , Tibshirani R . Regularization paths for generalized linear models via coordinate descent. J Stat Softw. 2010;33(1):1‐22. 20808728 PMC2929880

[16] R Core Team . *R: A Language and Environment for Statistical Computing*. R Foundation for Statistical Computing; 2021.

[17] Whitwell JL . Voxel‐based morphometry: an automated technique for assessing structural changes in the brain. J Neurosci. 2009;29(31):9661‐9664. 19657018 10.1523/JNEUROSCI.2160-09.2009 PMC6666603

[18] Carroll M , Cecchi G , Rish I , Garg R , Rao A . Prediction and interpretation of distributed neural activity with sparse models. NeuroImage. 2009;44(1):112‐122. 18793733 10.1016/j.neuroimage.2008.08.020

[19] Jiang W , Lakshminarayanan P , Hui X , et al. Machine learning methods uncover radiomorphologic dose patterns in salivary glands that predict xerostomia in patients with head and neck cancer. Adv Radiat Oncol. 2019;4(2):401‐412. 31011686 10.1016/j.adro.2018.11.008 PMC6460328

[20] Nabi R , McNutt T , Shpitser I . Semiparametric causal sufficient dimension reduction of high dimensional treatments. In: Proceedings of the 38th Conference on Uncertainty in Artificial Intelligence. PMLR; 2020:1445‐1455.

[21] Gilpin LH , Bau D , Yuan BZ , Bajwa A , Specter M , Kagal L . Explaining explanations: an overview of interpretability of machine learning. In: 2018 IEEE 5th International Conference on Data Science and Advanced Analytics (DSAA). IEEE; 2018:80‐89.

[22] Schultheiss TE , Orton CG , Peck RA . Models in radiotherapy: volume effects. Med Phys. 1983;10(4):410‐415. 6888354 10.1118/1.595312

[23] Withers HR , Taylor JM , Maciejewski B . Treatment volume and tissue tolerance. Int J Radiat Oncol Biol Phys. 1988;14(4):751‐759. 3350731 10.1016/0360-3016(88)90098-3

[24] Caron A , Baio G , Manolopoulou I . Sparse Bayesian Causal Forests for Heterogeneous Treatment Effects Estimation. 2021.

[25] Shelley L , Scaife J , Romanchikova M , et al. Delivered dose can be a better predictor of rectal toxicity than planned dose in prostate radiotherapy. Radiother Oncol. 2017;123(3):466‐471. 28460825 10.1016/j.radonc.2017.04.008 PMC5486775

[26] Veitch V , Sridhar D , Blei DM . Adapting Text Embeddings for Causal Inference. 2020.

