# Supplementary material for: Methods of causal effect estimation for high‐dimensional treatments: A radiotherapy simulation study
Source: Med Phys. 2025 Jun 2;52(7):e17919. doi: 10.1002/mp.17919 (PMC12258005; doi:10.1002/mp.17919)
Supplement: Supplementary file 1 — Supporting Information [file MP-52-0-s001.pdf]

Methods of Causal Effect Estimation for High-Dimensional Treatments: A Radiotherapy Simulation Study

Alexander Jenkins[1], Eliana Vasquez Osorio[2,3], Andrew Green[4], Marcel van Herk[2,3], Matthew Sperrin[5,+], Alan McWilliam[2,3,+]

[1] Imperial College London, Department of Electrical and Electronic Engineering, London, SW7 2AZ, United Kingdom
[2] University of Manchester, Division of Cancer Sciences, Manchester, M13 9PL, United Kingdom
[3] The Christie NHS Foundation Trust, Manchester, UK
[4] European Bioinformatics Institute (EMBL-EBI), Cambridge, UK
[5] University of Manchester, Division of Informatics, Imaging & Data Sciences, Manchester, M13 9PL, United Kingdom
[+] Joint senior authors

Version typeset June 2, 2025

Corresponding author: Dr Alan McWilliam
The Paterson Building
Wilmslow Road
Manchester
M20 4BX

alan.mcwilliam@manchester.ac.uk

I. Data-generating process

I.A. Treatment plans

The planned dose distribution is created in the style of VMAT treatment, where radiation is accumulated at the tumour by varying dose in a continual arc around the patient's tumour. VMAT is a cutting-edge treatment delivery technique which is used for most patient treatments. Because VMAT is delivered in an arc around the patient, clinicians cannot precisely control the dose at the individual voxel-level. Instead, clinicians parameterise the dose distribution in the tumour and OAR regions. For example, by controlling the mean and/or variance of dose in a region. An optimiser then constructs a planned dose distribution which satisfies these constraints. We consider three clinical intervention parameters in this work: the variance of dose around the tumour, V_T, the magnitude of dose at the OAR, M_O, and variance of dose around the OAR, V_O. An additional clinical parameter we consider is the prescribed dose to the tumour, M_T. This parameter is not controlled during treatment planning, as it is prescribed beforehand for curative intent. Therefore, an arbitrary constant positive value of $M_T = 4.0$ will be set for all simulated patients in this work.

VMAT treatment is typically delivered in fractions, i.e. over multiple treatment days, to increase treatment effectiveness. In this work, a single treatment fraction is simulated, but the presented methods can be easily generalised to multiple fractions. To spare the dose to the OAR during VMAT treatment, the optimisation reduces the dose passing through this location. We refer to this as the *organ ray*. The organ ray is defined as a straight line toward the tumour, that passes through the OAR position and to the edge of the grid.

The planned dose distribution is simulated as

$$P(x, y) = 2 + f_{D_T}(x, y) - f_{D_O}(x, y). \tag{1}$$

The constant term ensures a non-zero dose around the body. The second term is the function describing the dose around the tumour

$$f_{D_T}(x, y) = M_T \exp\left(\frac{-((x - T_x)^2 + (y - T_y)^2)}{V_T}\right), \tag{2}$$

where T_x and T_y are the x- and y-coordinates of the patient's tumour. The final term controls for the dose around the organ ray, and it is given by

$$f_{D_O}(x, y) = M_O \exp\left(\frac{-d_{ray}(x, y)^2}{V_O}\right), \tag{3}$$

where $d_{ray}(x,y)$ is the Euclidean distance of point (x,y) from the closest point on the organ ray.

In VMAT treatment plans, if the dose is reduced along the organ ray, then it must be increased elsewhere to ensure M_T is delivered to the tumour. To ensure this in our simulation, we take the total dose subtracted along the organ ray, $\int\int f_{D_O}(x,y)dxdy$, and add it homogeneously across the dose distribution by increasing V_T.

I.B. Treatment uncertainties

Due to different sources of random error present in treatment delivery, the planned dose distribution and the delivered dose distribution vary. In our simulation, we take into account different sources of random error. We consider that the delivered dose distributions are simulated with realistic treatment uncertainties operating at three different scales: dosimetric uncertainty, anatomical motion, and setup uncertainty.

Dosimetric uncertainty is represented as independent noise at each pixel (i,j), where $i \in [1,...,N_x]$ and $j \in [1,...,N_y]$, and N_x and N_y are the number of pixels in x and y. This will be sampled from a mean-zero normal distribution, $U_{D_{ij}} \sim N(0,\sigma_D^2)$, where σ_D^2 controls the variance. The anatomical motion will be simulated as spatially correlated noise sampled from a mean-zero Gaussian process

$$\mathbf{M} = M_{GP} \times GP(0, K(x,x')), \tag{4}$$

where M_{GP} controls the magnitude of the noise, and $K(x,x')$ is a 2D radial basis function kernel parameterised by σ_{GP}^2; a parameter that controls the amount of correlation between two adjacent pixels. Finally, the setup uncertainty is represented as random shifts in the x- and y-coordinates of the entire planned dose distribution, i.e. $P(x+L_x, y+L_y)$, where $L_x, L_y \sim N(0,\sigma_L^2)$ are sampled from the same distribution with σ_L^2 controlling the variance. The parameters for these distributions will be varied in logarithmic scale to demonstrate their influence.

Therefore, the delivered dose distribution at pixel location $(x,y) = (i,j)$ will be generated as

$$D_{ij} = P(i+L_x, j+L_y) + M_{ij} + U_{D_{ij}}, \tag{5}$$

where the function $P(\cdot)$ is given by (1) and M_{ij} is given by (4) sampled at pixel location $(x, y) = (i, j)$.

I.C. Form of the SCM

Each of the variables in the DAG of this simulation, as shown in Figure 2 of the Main Manuscript text, are generated as a function of their parents and independent noise, i.e. using a SCM. The functions that will be used to generate each variable are defined as

$$C = U_C, \tag{6a}$$

$$V_T = \frac{6(C+3)^2}{5} + 3 + U_{V_T}, \tag{6b}$$

$$V_O = (1 + \frac{U_{V_O}(3+C)}{15}) \times s(O_{x,y}, T_{x,y})^2, \tag{6c}$$

$$M_O = (\frac{3+C}{15} f_{D_T}(O_x, O_y) + U_{M_O}) \times (\frac{1}{2} + s(O_{x,y}, T_{x,y})), \tag{6d}$$

$$Y = 5C + U_Y + \sum_{i=1}^{N_x} \sum_{j=1}^{N_y} \left(D_{ij}\theta_{ij} + \frac{C}{2}(D_{ij}\xi_{ij}) \right). \tag{6e}$$

Unless otherwise stated, the random noise for each variable k in (6), U_k, will be sampled independently from a mean-zero Gaussian distribution with unit variance, $U_k \sim N(0, 1)$. In (6c) and (6d), $s(O_{x,y}, T_{x,y}) = \frac{\sqrt{(T_x - O_x)^2 + (T_y - O_y)^2}}{\sqrt{(T_x + O_x)^2 + (T_y + O_y)^2}}$ is a non-linear function that scales V_O and M_O depending on the relative x- and y-coordinates of the organ, O_x, O_y, and the tumour, T_x, T_y. This scaling is used in (6c) and (6d) such that if the tumour is closer to the organ, the amount of dose spared to the organ is reduced and the fall-off is sharp. The function $f_{D_T}(\cdot)$ is defined in (2).

I.C.1. Average Treatment Effect and its Variance

The ATE at each pixel, ij, can be written using the concept of potential outcomes:

1. Define Y^0 as the potential outcome if a single patient receives no treatment at all pixels, i.e. $D_{ij} = 0 \quad \forall i, j$.

2. Define Y^1_{ij} as the potential outcome if patient receives treatment $D_{ij} = 1$ at pixel ij, and zero treatment at all other pixels, $D_{kl} = 0 \quad \forall k, l \neq i, j$.

The treatment effect for a single patient at pixel ij is then, $TE_{ij} = Y^1_{ij} - Y^0$. However, we cannot directly observe both potential outcomes for the same patient. Instead we compute the ATE at pixel ij as

$$\text{ATE}_{ij} = \mathbb{E}[Y^1_{ij}] - \mathbb{E}[Y^0], \tag{7}$$

where the expectation is over patients.

Substituting (6e) into (7), we get for the first term

$$\mathbb{E}[Y^1_{ij}] = \mathbb{E}[5C + U_Y + \theta_{ij} + \frac{C}{2}\xi_{ij}]. \tag{8}$$

Since C, U_Y are independent random variables with $\mathbb{E}[C] = \mathbb{E}[U_Y] = 0$, and θ_{ij} is constant, this simplifies to

$$\mathbb{E}[Y^1_{ij}] = \theta_{ij}. \tag{9}$$

For the second term in (7), we get

$$\mathbb{E}[Y^0] = \mathbb{E}[5C + U_Y] = 0. \tag{10}$$

Therefore, the ATE at pixel ij can be written as

$$\text{ATE}_{ij} = \theta_{ij}. \tag{11}$$

Similarly, the Conditional Average Treatment Effect (CATE) for the value $C = 0$, can be found to be

$$\text{CATE}_{ij}|C = 0 = \mathbb{E}[Y^1_{ij}|C = 0] - \mathbb{E}[Y^0|C = 0] = \theta_{ij}. \tag{12}$$

I.C.2. Variance of Treatment Effects

The treatment effect at pixel ij can be written as

$$\text{TE}_{ij} = Y^1_{ij} - Y^0 = 5C + U_Y + \theta_{ij} + \frac{C}{2}\xi_{ij} - (5C + U_Y) = \theta_{ij} + \frac{C}{2}\xi_{ij}. \tag{13}$$

The variance of the treatment effect can be computed as

$$\sigma^2_{ij} = \text{Var}(TE_{ij}) = \text{Var}(\theta_{ij} + \frac{C}{2}\xi_{ij}). \tag{14}$$

From the definition of variance, $\text{Var}(X) = \mathbb{E}[(X - \mathbb{E}[X])^2]$, we substitute (14) and (11) to get

$$\sigma^2_{ij} = \mathbb{E}[(\theta_{ij} + \frac{C}{2}\xi_{ij} - \theta_{ij})^2] = \mathbb{E}[(\frac{C}{2}\xi_{ij})^2]. \tag{15}$$

Since ξ_{ij} is constant and $\text{Var}(C) = \mathbb{E}[C^2] - \mathbb{E}[C]^2 = \mathbb{E}[C^2] = 1$, this simplifies to

$$\sigma_{ij}^2 = (\frac{\xi_{ij}}{2})^2 \mathbb{E}[C^2] = \frac{\xi_{ij}^2}{4}. \tag{16}$$

The treatment effect conditioned on $C = 0$ is calculated by substituting $C = 0$ into (13), to give

$$TE_{ij}|C = 0 = \theta_{ij}. \tag{17}$$

Therefore its variance is

$$\sigma_{ij|C=0}^2 = \text{Var}(\theta_{ij}) = 0 \tag{18}$$

since θ_{ij} is a constant.

I.D. The estimand and interaction strength arrays

The continuous outcome, Y, for each patient, will be generated using (6e). The ATE of D_{ij} on Y is linear and parameterised in expectation by θ_{ij}. An interaction effect between D_{ij} and the covariate C, on Y, will also be included in the simulation to represent the realistic dependency of dose on a measured confounder (e.g. age). This is also linear and is parameterised by ξ_{ij}. The estimand array, $\boldsymbol{\theta}$, will be generated using $f(x, y)$ evaluated at the x- and y-coordinates of each pixel, in any required resolution,

$$\begin{aligned} g(x, y) &= 3 \sin \left(\frac{10x}{N_x} + \frac{3}{2} \sin \left(\frac{10y}{N_y} \right) - 0.5 \right), \\ f(x, y) &= \begin{cases} g(x, y), & \text{if } g(x, y) > 0 \\ 0, & \text{otherwise.} \end{cases} \end{aligned} \tag{19}$$

The function, $f(x, y)$, represents the spatial dose response and produces values in the range $[0, 3]$. The estimand array, $\boldsymbol{\theta} = \{\theta_{ij} | i \in [1, ..., N_x], j \in [1, ..., N_y]\}$, where $\theta_{ij} = f(i, j)$, will be constant for all patients, without loss of generality. The form of $f(x, y)$ in (19) is chosen such that the dose response is spatially complex, inhomogeneous, positive (dose always increases the outcome value/odds), and sparse (zero effect in many pixels).

The interaction strength between D_{ij} and the covariate C, on Y, will be defined by the following function, $z(x, y)$, which is evaluated at the x- and y-coordinates of each pixel to

define the interaction strength array, $\boldsymbol{\xi}$, in any required resolution,

$$
\begin{aligned}
h(x,y) &= 3\sin\left(\frac{xy}{20N_y}\right),\\
z(x,y) &= \begin{cases} h(x,y), & \text{if } h(x,y) > 0 \text{ and } g(x,y) > 0\\ 0, & \text{otherwise,}\end{cases}
\end{aligned} \tag{20}
$$

where $g(x,y)$ is the function defined in (19). The function, $z(x,y)$, produces values in the range $[0,3]$. The interaction strength array, $\boldsymbol{\xi} = \{\xi_{ij} | i \in [1,...,N_x], j \in [1,...,N_y]\}$, where $\xi_{ij} = z(i,j)$, will be constant for all patients, without loss of generality. The form of $z(x,y)$ in (20) is chosen such that the interaction is spatially complex, inhomogeneous, positive, and is non-zero only in regions where the dose response, $\boldsymbol{\theta}$, is non-zero.

II. Baseline estimators

To establish a baseline for comparison with our proposed estimators, an additional causal inference based estimator, a causal regression (without sparsity), will be evaluated. In addition, the current voxel-based estimators in radiotherapy, the pixel-wise univariate linear regression, will be tested.

The causal regression (without sparsity) is defined as a linear regression using the identified total set of features. Two variants of the pixel-wise univariate linear regression will be tested. Specifically, the following two models will be applied to each pixel $i \in [1,...,N_x]$ and $j \in [1,...,N_y]$:

$$Y = \beta_0 + \hat{\theta}_{ij}^{PP} P(i,j); \tag{21a}$$

$$Y = \beta_0 + \hat{\theta}_{ij}^{PD} D_{ij}. \tag{21b}$$

The equation in (21a) is an estimation method which uses the planned dose at the pixel, $P(i,j)$, as calculated from (1). The fitted gradient, $\hat{\theta}_{ij}^{PP}$, is interpreted as the estimate of the ATE of the delivered dose at that pixel. The estimation method in (21a) will be referred to as *pixel-wise planned*, hence the PP notation. The equation in (21b) is an estimation method which uses the delivered dose at the pixel, D_{ij}, as calculated from (6e). The fitted gradient, $\hat{\theta}_{ij}^{PD}$, is interpreted as the estimate of the ATE of the delivered dose at that pixel. The estimation method in (21b) will be referred to as *pixel-wise delivered*, hence the PD notation. In both (21a) and (21b), β_0 is the intercept of the model.

III. Statement and Relation to Prior Work

Compared to the master's thesis[1] of the lead author, our current manuscript includes several substantive extensions:

- Extended estimands and results: While the thesis focused solely on CATE(C=0), our manuscript aligns the ATE and CATE(C=0) to give the same value in expectation. This demonstrates our estimator's utility for two different important estimands in causal inference literature. All experiments have been re-run for these new estimands, with updated results and interpretations.
- New theoretical contributions: We have derived the variance of both the ATE and CATE(C=0), as shown in Supplementary Materials I.C.1 and I.C.2. These important theoretical results aid readers in understanding the methodology and the performance differences that would be expected depending on the estimand definition.
- Substantial manuscript revision: The text has been significantly re-written and re-structured compared to the thesis chapter it was originally based on, providing clearer explanations, improved contextual framing, and more thorough interpretation of results.

References

[1] A. L. Jenkins, Voxel-Based Causal Inference in Radiotherapy: A Simulation Study, Master's thesis, University of Manchester, 2021.
